# Potential Applications of Microparticulate-Based Bacterial Outer Membrane Vesicles (OMVs) Vaccine Platform for Sexually Transmitted Diseases (STDs): Gonorrhea, Chlamydia, and Syphilis

**DOI:** 10.3390/vaccines9111245

**Published:** 2021-10-27

**Authors:** Christiane Chbib, Sarthak M. Shah, Rikhav P. Gala, Mohammad N. Uddin

**Affiliations:** 1 Department of Pharmaceutical Science, College of Pharmacy, Larkin University, Miami, FL 33169, USA; cchbib@ularkin.org; 2 Department of Pharmaceutical Sciences, College of Pharmacy, Mercer University, Atlanta, GA 30341, USA; sarthak.modi.shah@live.mercer.edu; 3 Fraunhofer USA, Center Mid-Atlantic, Biotechnology Division, Newark, DE 19702, USA; Rgala@fraunhofer.org

**Keywords:** antibiotic, chlamydia, gonorrhea, syphilis, sexually transmitted diseases (STDs), microparticulate, vaccine, outer membrane vesicles (OMV)

## Abstract

Sexually transmitted diseases (STDs) are a major global health issue. Approximately 250 million new cases of STDs occur each year globally. Currently, only three STDs (human papillomavirus (HPV), hepatitis A, and hepatitis B) are preventable by vaccines. Vaccines for other STDs, including gonorrhea, chlamydia, and syphilis, await successful development. Currently, all of these STDs are treated with antibiotics. However, the efficacy of antibiotics is facing growing challenge due to the emergence of bacterial resistance. Therefore, alternative therapeutic approaches, including the development of vaccines against these STDs, should be explored to tackle this important global public health issue. Mass vaccination could be more efficient in reducing the spread of these highly contagious diseases. Bacterial outer membrane vesicle (OMV) is a potential antigen used to prevent STDs. OMVs are released spontaneously during growth by many Gram-negative bacteria. They present a wide range of surface antigens in native conformation that possess interesting properties such as immunogenicity, adjuvant potential, and the ability to be taken up by immune cells, all of which make them an attractive target for application as vaccines against pathogenic bacteria. The major challenge associated with the use of OMVs is its fragile structure and stability. However, a particulate form of the vaccine could be a suitable delivery system that can protect the antigen from degradation by a harsh acidic or enzymatic environment. The particulate form of the vaccine can also act as an adjuvant by itself. This review will highlight some practical methods for formulating microparticulate OMV-based vaccines for STDs.

## 1. Introduction

According to the Center of Disease Control (CDC), several STDs that are still of concern to public health and safety include bacterial vaginosis, chlamydia, gonorrhea, viral hepatitis, genital herpes, HIV/AIDS, HPV, pelvic inflammatory disease (PID), syphilis, trichomoniasis, chancroid, and scabies [1]. Most STDs are caused by Gram-negative bacterial infection and transmission, specifically, gonorrhea, chlamydia, and syphilis [1,2,3]. STDs are highly contagious and are passed along to other individuals via vaginal, oral, or anal sex [1].

In this review, we aim to summarize the recent advances on OMVs as vaccine candidates against STDs, with special emphasis on Chlamydiae trachomatis, Treponema pallidum, and Neisseria gonorrhoeae.

Chlamydia is considered one of the most common STDs in both men and women. In a recent study conducted by CDC, many cases of chlamydia in the United States were reported between 2015 and 2019. During this period, a significant increase in chlamydia infections was observed from 2015 (1,526,658 cases) to 2019 (1,808,703 cases) [4]. Chlamydia is a common STD caused by Chlamydia trachomatis [5]. Women infected may experience pain during intercourse, a burning sensation when urinating, and abnormal vaginal discharge. Men may experience symptoms such as a burning sensation when urinating, itching or burning around the opening of the penis, and pain and swelling of the testicles [5,6]. Chlamydia is currently treated with antibiotics; however, this approach appears to be less effective in reducing the prevalence of this infection. Therefore, the development of a vaccine might hold promise in preventing this disease, resulting in a reduced number of cases.

Syphilis is another commonly occurring STD caused by the spirochete bacterium Treponema pallidum. In 2019, 129,813 cases of syphilis were reported in the United States. This number is less compared to 616,392 cases of gonorrhea reported that year, but is significantly higher than 37,968 new diagnoses of HIV infection [7] that year. The CDC reported that the number of individuals diagnosed with syphilis is increasing. Congenital syphilis, where the bacterium is passed from pregnant women to their babies, continues to be a public health concern. In 2019, approximately 1900 new cases of congenital syphilis were reported [8]. Syphilis is transmitted between individual by direct contact during anal, vaginal, or oral sex, specifically when an individual comes in direct contact with a syphilitic sore known as a chancre. This disease is highly contagious, and because its transmission occurs by contact with primary chancres or via secondary lesions, prevention of this disease would be the most effective way to reduce the number of cases. In this scenario, successful development of a vaccine could be the most effective way for controlling this disease [8,9].

Gonorrhea, which is also a common STD, is caused by Gram-negative diplococcus bacteria, Neisseria gonorrhoeae. In 2019, a total of 616,392 cases of gonorrhea were reported to the CDC. This was the second-most common notifiable conditions in the United States for that year, with an uprise of a staggering 92.0% since their historic low in 2009. During 2018–2019, the overall rate of reported gonorrhea increased 5.7%. Rates of reported cases increased across different regions, gender, race, and ethnicity. Since 2013, the rate of gonorrhea infection has been found to be higher in men compared to women. Among men, the rate of reported gonorrhea increased 5.9% during 2018–2019 and 60.6% during 2015–2019. Rates among women increased 5.1% during 2018–2019 and 43.6% during 2015–2019 [7]. Currently, gonorrhea is treated and cured with antibiotic therapy, but the emergence of multi-drug resistant gonorrhea poses an important challenge for future treatment options. A vaccine could be a more effective way to prevent the disease and reduce its number of cases.

As discussed before, the current treatment for gonorrhea, chlamydia, and syphilis are solely dependent on antibiotic therapy. However, there is a growing concern about the future of antibiotic therapy for these diseases due to the emergence of bacterial resistance to one or multiple classes of antibiotics. This may leave us with a few or no options for effective antibiotic therapy for those diseases. To reduce the incidence of resistance organisms, the CDC updated its guidelines to increase the dose of these drugs. In 2010, they provided guidelines for the treatment of uncomplicated gonococcal infections of the urethra, rectum, and cervix by a one 250 mg intramuscular (IM) dose of ceftriaxone and one 1 g oral dose of azithromycin [10]. However, the guidelines for treating gonococcal infections changed ten years later in 2020, by increasing the ceftriaxone (IM) dose from 250 mg to 500 mg. The CDC acknowledged that the effectiveness of this treatment regime may dwindle due to the increase in gonorrhea’s ability to acquire antimicrobial resistance at an alarming rate [10]. The rise in resistance indicates that the treatment guidelines may change again in the future to combat STDs effectively. Another possible way to treat these STDs can be accomplished by formulating a microparticulate-based bacterial outer membrane vesicle (OMV) vaccine. This review discusses the structure and composition of OMVs, the potential of OMVs as vaccine platforms, and highlight some practical methods for formulating a particulate vaccine for the prevention or treatment of STDs.

## 2. Structure, Function, and Composition of OMVs

OMVs are spherical vesicles with a size of 20–250 nm produced naturally by Gram-negative bacteria. The vesicles are formed by lipid bilayer membranes derived from the bacterial outer membrane [11,12,13]. OMV consists of a wide variety of bacterial-derived products such as enzymes, antigens, virulence factors, and pathogen-associated molecular patterns (PAMPs) such as lipopolysaccharides, DNA, RNA, peptidoglycans, and others [14]. Bacteria may produce OMVs, bringing about favorable changes within their environment that promote their growth and survival [15,16]. Research shows that OMVs could contain virulence factors and modulate the host immune system during pathogenesis while helping them with nutrient acquisition, ecological niche safeguarding [17,18], and providing structural support through biofilm formation [19,20].

**Figure 1:**
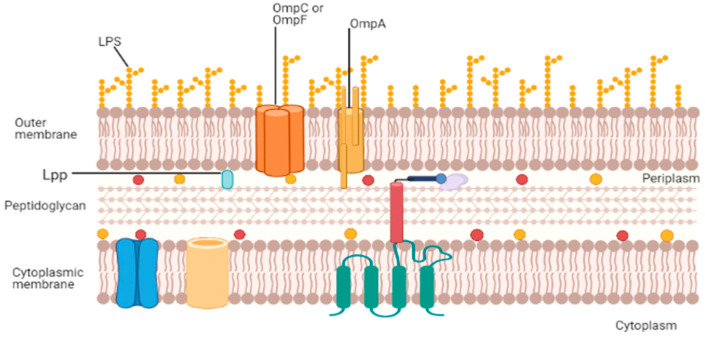
Composition of Gram-negative cell envelope [12]. LPS: lipopolysaccharides, Lpp: Braun’s lipoprotein, and OmpC/F/A: outer-membrane protein.

The cell envelope of Gram-negative bacteria (Figure 1) is the primary source of OMVs. It consists of two membranes: the outer membrane and the cytoplasmic membrane. The space between each membrane is known as periplasmic space, consisting of a layer of peptidoglycan (PG) with periplasmic proteins [15]. The outer membrane is usually formed of phospholipids and lipopolysaccharides (LPS), also known as endotoxins. The cytoplasmic membrane comprises a phospholipid bilayer, which acts as an electrochemical barrier [15]. LPS is composed of a lipid, a core made of oligosaccharides, and an antigen.

**Figure 2:**
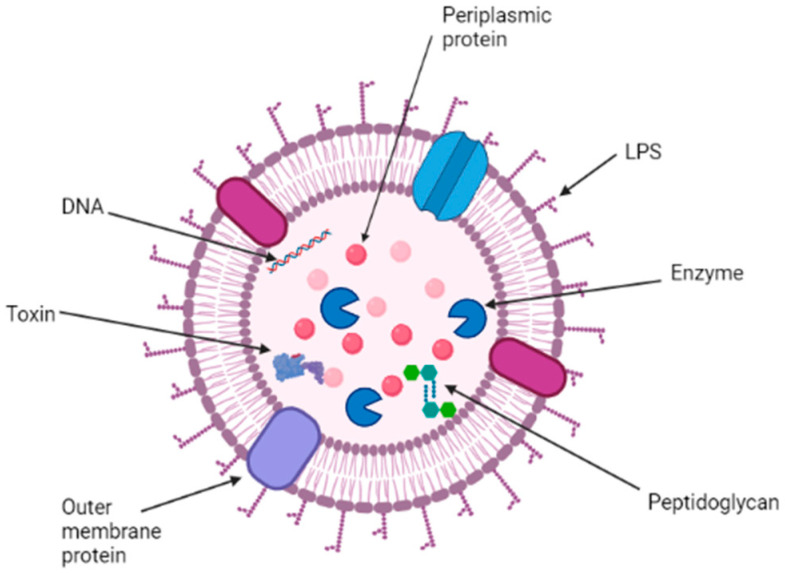
Structure and content of Gram-negative bacterial outer membrane vesicle (OMV) [21].

OMVs are spherical portions (~20–250 nm in diameter) of the outer membrane, comprising periplasmic luminal elements with the ability to bud and detach from the cell during active growth [16]. Therefore, the biogenesis of OMVs depends on the dissociation of the outer membrane from the underlying PG in areas, followed by a split without affecting the envelope integrity (Figure 2) [16,17,18,19,20].

Multiple factors influence the biogenesis of the OMVs in bacteria. OMV production is enhanced in areas with reduced Braun’s lipoprotein (Lpp) and prostaglandin crosslinks. In places where misfolded proteins accumulate, crosslinks can be relocated or depleted, leading to outer membrane bulging, increasing OMV production [12]. OMVs play essential roles in bacterial physiology. While stress conditions enhance OMV production, an OMV removes toxic compounds responsible for creating stress. OMVs are also a good source of carbon and nitrogen and provide the bacterial cell with essential nutrients by breaking down macromolecules. Additionally, OMVs hold iron and zinc acquisition systems that allow bacteria to extract these essential metals for survival [12]. OMVs play essential roles in biofilm formation, regulating bacterial virulence and drug resistance [22,23].

## 3. Isolation of OMVs

OMVs are naturally produced from the bacterial cell surface. They are concentrated in the cell-free supernatant of bacterial cultures. To isolate OMVs, different methods can be applied to purify crude OMVs. Analysis methods include protein profiling, detection of indicator proteins (immunoblot analysis), lipid profiling (lipid extraction and LC-MS analysis), vesicle size determination, a rough estimation of biomass, and quantifications of defined OMV components [24,25,26,27,28].

OMVs that are obtained for vaccine formulations are of different types. The first type is spontaneous “sOMVs”, which are produced naturally from the budding of bacteria. The second type of OMVs is detergent “dOMVs”, produced after a selected detergent is applied to the bacterium. The third type is native “nOMVs”, which are produced using nondetergent methods such as sonication. Immunologically, OMVs produced from the bacterium via a detergent intervention are different from OMVs made from sOMVs and nOMVs [29].

## 4. OMVs as a Vaccine Candidate

OMVs as a vaccine tool have been researched for many years [30]. Recently, the use of OMVs against *Neisseria meningitidis* was assessed [31]. It was shown that the complex formed from the adjuvant lipopolysaccharide (LPS) and bacterial surface antigen allows OMVs to generate an adaptive immune response (Figure 2). This result raises the possibility that the OMVs could be used as a vaccine if we can overcome its inherent limitation of instability.

OMVs, which are spontaneously released by many Gram-negative bacteria, possess immunogenicity, adjuvant potential, and the ability to be taken up by immune cells. These features make them an attractive target for application as vaccines against pathogenic bacteria. By definition, a vaccine is a pharmaceutical product that stimulates the immune system to prevent pathogens from causing disease. To elicit an immune response specific for a pathogen, a vaccine product should resemble a pathogen without causing any disease associated with an antigen [32]. From a vaccine development standpoint, a vaccine candidate ideally should have a size similar to that of the natural antigen. The vaccine candidate should also contain a pathogen-associated molecular pattern. In contrast, there are several unwanted properties that a vaccine candidate should not possess. Examples of such unwanted properties include creating an enormous variety of specific surface components, mimicry with host components, production of proteases that degrade antibodies, or development of biofilms. These are not desired in a vaccine candidate, as they can cause the pathogens to develop various immune evasion strategies.

Considering the availability of desirable features, OMVs are incorporated in vaccine formulations. OMVs have a proper size (20–200 nm) to enable their entry into lymphatic vessels and subsequent uptake by antigen-presenting cells [8]. This size resembles the size of a virus with a range of 20 to 300 nm [12]. Due to the size of OMVs, they are identified as foreign matter in the body and trigger an immune response. This size similarity enables OMVs to enter lymphatic vessels and be taken up by antigen-presenting cells [33]. OMVs also contain natural components that stimulate humoral and cell-mediated immune responses, since they resemble the bacterial antigenic surface of the pathogen [34]. One potential advantage of their use in vaccine formulations is that because OMVs do not have a series of other host proteins, chances for potential side effects are avoided [34].

Recent studies have reported specific characteristics of OMVs in different Gram-negative bacteria. The OMV of Chlamydia trachomatis is consistent with the general structure in Figure 2. Recent studies have shown that HtrA, a highly conserved protein, is released in the cytoplasm of chlamydial-infected cells. It has been shown to play a major role in the infection pathology [35]. Chlamydia has an outer membrane that contains the typical lipopolysaccharide and proteins but lacks peptidoglycan layer, which makes it different from other Gram-negative bacteria [36].

The peptidoglycan layer of Treponema pallidum is chemically different, thinner, and further distal from the outer membrane when compared to the typical Gram-negative bacteria in Figure 2. It does contain a minor amount of lipoprotein but lacks lipopolysaccharides [37] in addition to the structural feature of Tp0624, which prevents it from binding to typical peptidoglycan [38]. On the other hand, the lipopolysaccharide is found to be the most abundant on the surface of a gonococcal cell. It is proven to contribute to the Neisseria gonorrhoeae pathogenesis [39]. Those differences in the OMV structures of the three pathogens are the reason why OMV-based vaccine development and advances will be different but specific to each pathogen.

## 5. Updates on the Pre-Clinical and Clinical Study on OMV-Based Vaccine Development

Chlamydia trachomatis: Bartolini et al. described that *C. trachomatis* HtrA can be delivered to the OMV compartment, which stimulates antibodies. This is in line with the theory that OMVs carry the majority of the PAMPs found in bacteria. Additional studies are required to assess the level of HtrA protein in OMVs as well as the productive yield of OMV [40]. Currently, one intranasal major outer membrane protein vaccine is being tested pre-clinically as a nano-emulsion [41], but no official results are published assessing the success of this method.

Treponema pallidum: The recent use of M131 as OMV in rabbits to test its immunogenicity showed that the phosphorylcholine surface target of M131 can play a key role for vaccine development [42]. This discovery may serve as a major step towards OMV vaccine development for syphilis, especially after the challenges faced during isolating OMV in Treponema pallidum. More studies are still needed to prove the immunogenicity of M131.

Neisseria gonorrhoeae: Currently, two OMV vaccines exist for Neisseria meningitis (Nm). The impact of the Nm OMV vaccine on Neisseria gonorrhea (Ng) has shown potential cross-reaction [43]. A marked decline in the number of cases of gonorrhea was reported in Cuba following the implementation of the OMV vaccine for meningitis. On the other hand, the number of incidents were rising for the other STDs such as syphilis and genital warts [44]; the same trend was observed in New Zealand [45]. It has been hypothesized that the Nm OMV vaccine could offer protection against gonorrhea. This hypothesis was tested by Petousis-Harris et al. [46], where patients between the ages of 15 and 30 years with a confirmed gonorrhea case were included in the trial. Results showed that patients who received the vaccine had a lower incidence of gonorrhea. Subsequently, another study reported the effectiveness of the vaccine on gonorrhea-associated hospitalization. To understand this cross protection, Davenport et al. [47] shared some pre-clinical evidence that OMV vaccine can reprogram the mucosa area, which allows the cross-protection between Nm and Ng. While the findings are encouraging, rigorous studies will be required in the future to confirm the efficacy of this vaccine.

Although OMVs are potential vaccine candidates, they have some reported limitations that need to be addressed for successful vaccine development. Some of the limitations are (i) the high reactogenicity of pathogen-associated molecular patterns such as LPS, (ii) low expression levels of relevant protective antigens, (iii) strain variation resulting in many subtypes of specific antigens, thus lower coverage, (iv) immuno-dominant antigens that misdirect the immune response, and (v) molecules, which are immunosuppressive or otherwise interfere with a protective immune response. Therefore, genetic engineering of the OMV-producing strain should be applied to overcome those shortcomings by removing, adding, or altering OMV proteins and other components. In addition, preparing a biodegradable and biocompatible polymer-based particulate form of the OMV vaccine can also address this issue [32]. Another approach could be structural modifications to OMV. Kyu-Tae Chang et al. developed a safe and highly effective vaccine delivery system in which OMVs were modified to have properties of intrinsically low endotoxicity sufficient for the delivery of foreign antigens. Their strategy involved mutational inactivation of the MsbB (LpxM) lipid A acyltransferase to generate OMVs of reduced endotoxicity from *Escherichia coli* (*E. coli*) O157:H7. The results suggest that by using genetic engineering-based approaches, the native OMVs could be modified to have both intrinsically low endotoxicity and a foreign epitope tag to establish a platform technology for developing multifunctional vaccine delivery vehicles [48,49,50,51].

## 6. Particulate Delivery System of Outer Membrane Vesicles (OMVs)

Although OMV technology is being tested for its use as a vaccine, the structure of OMV is very fragile and vesicle-like, making it unstable. An antigen must keep its structural integrity before uptake by antigen-presenting cells. The destruction of the structure of the antigen before uptake may not trigger immunogenicity. The stability OMVs could be improved by developing a particulate form of vaccine using a biodegradable and biocompatible polymer. The particulate form of a vaccine offers several advantages. Particles can be used as antigen carriers and/or adjuvant in the same preparation [52]. Particulate carriers can also serve as effective antigen delivery systems that can enhance and/or facilitate the uptake of antigens by antigen-presenting cells [53,54]. In addition, the particle can be used as a depot for the controlled release of antigen, thereby increasing the availability of the antigen to the immune cells for a more extended period [55,56]. It can hold more than one antigen and/or adjuvants when needed. Particle formulations possess the ability to modulate the type of immune responses induced when used alone or in combination with other immune-stimulatory compounds [57]. Particulates can protect the integrity of antigens against degradation especially in the harsh acidic conditions of the stomach and enzymatic degradation in the GI tract [58,59]. In the case of OMV, the most important challenge is to preserve the integrity of the structure. Thus, a polymeric particulate formulation can protect the fragile structure of OMV.

Recently, polymeric particle formulations have gained great attention in the field of vaccine delivery research due to their potentially advantageous biocompatibility and biodegradability [60]. Several natural and synthetic polymers such as polysaccharides [61], poly(D,L-lactic-coglycolic acid), (PLGA) poly(lactic acid) (PLA) [62], and poly(D,L-lactide-co-glycolide) (PLG) [63] have been used to make particles for vaccine delivery. These particles are able to either entrap or adsorb the antigen for delivery to specific cells or allow for sustained antigen release over time because of their slow biodegradation rate [64,65]. The size of OMV ranges from 30 nm to 70 nm, which may be ideal to prepare nanoparticulate formulation of vaccine delivery system. The unique physicochemical properties of nanoparticles include higher surface-to-volume ratio, small size, ability to encapsulate various drugs, and tunable surface chemistry, which give them many advantages over their bulk counterpart. There are also other biological advantages that include multivalent surface modification with targeting ligands, efficient navigation of the complex in an in vivo environment, increased intracellular trafficking, the addition of any charged particles to increase target selectivity, and sustained release of the drug. Together, these advantages make nanoparticles ideal candidates for formulating drugs for the most prevalent and challenging diseases [66,67].

Recently, the integration of synthetic nanoparticles (NPs) and natural cellular materials such as cell membranes or exosomes has led to the creation of various biomimetic nanoparticles that can be applied to OMVs [68,69]. OMVs generally have a hollow, vesicular nanostructure composed of phospholipids and lipopolysaccharide (LPS) that contain outer membrane proteins (OMPs) [70]. To improve their stability, G. Wu et al. [71] attempted to reinforce the OMVs structure by depositing the hollow-structured OMVs onto bovine serum albumin (BSA) NPs (BN). BSA protein has a high affinity to complexes with lipid molecules through hydrophobic interaction [71]. Another study reported the development of a novel strategy to engineer protein NPs stabilized by the intermolecular disulfide network [71]. The method employed by G. Wu et al. was green synthetic, easily accessed, and size controlled. Therefore, it was selected to be employed in the development of OMVs nano-vaccine formulation. The goal was to reinforce the OMV structure and uniform their size to prepare membrane-coated BSA nanoparticulate OMVs. The fragile structure of OMVs was observed to be reinforced internally by size-controlled BSA nanoparticles to obtain uniform and stable vaccines through hydrophobic interactions. The result showed that the BSA-OMV nanoparticles (BN-OMVs) were homogenous with a size around 100 nm and exhibited a core-shell structure. In the in vivo study, the subcutaneous BN-OMVs vaccination had remarkably higher specific antibody titers. This study demonstrated that the structure optimization improved the immune efficacy of OMVs for vaccine development [71].

## 7. Conclusions

STDs present a significant health issue worldwide. Three of the most common STDs are gonorrhea, chlamydia, and syphilis. Currently, only antibiotics are available to treat these diseases therapeutically. However, due to bacterial resistance, the effectiveness of therapeutic antibiotics is decreasing. Therefore, a preventive vaccination approach could be a good alternative to reduce the number of cases caused by STDs. OMV can be a good antigen candidate for vaccine against STDs due their size, immunogenicity, adjuvant potential, and the ability to be taken up by immune cells. The OMV-based vaccination concept is further supported by the pre-clinical and clinical study report. However, the major problem with OMV is its fragile structure. The particulate form of vaccine can address this structure related stability issue. Therefore, the particulate form of OMV can be a good choice for OMV vaccine delivery, which can increase its stability in the system by protecting it from acidic or enzymatic degradation. We also argue that the proposed particle-based OMV vaccine shelf life is expected to be several folds higher than that of conventional vaccines, since it is kept well protected from moisture. More research is needed to investigate the particulate form of OMV vaccines prepared for characterization and in-vitro evaluation.

## Data Availability

Data are available in correspondent journal.
